# Supplementary material for: Human Biomonitoring Data in Health Risk Assessments Published in Peer-Reviewed Journals between 2016 and 2021: Confronting Reality after a Preliminary Review
Source: Int J Environ Res Public Health. 2022 Mar 13;19(6):3362. doi: 10.3390/ijerph19063362 (PMC8955248; doi:10.3390/ijerph19063362)
Supplement: Supplementary file 1 [file ijerph-19-03362-s001.zip › ijerph-1629006-supplementary.pdf]

Human biomonitoring data in health risk assessments published in peer-reviewed journals between 2016 and 2021: Confronting reality after a preliminary review

Supplementary file

Appraisal tool

**Appraised publication:** e.g. TITLE					
Appraisal tool					
HRA element		**Appraisal question**	**Answer (Yes/No)***	**HBM****	Comments
Assessment context of HRA (assessment context answers the following key questions: what is to be assessed, why is to be assessed, which assessment endpoint is relevant, assessment timeframe; it is more specific than the general context of the publication)		Does the assessment clearly identify what is assessed and why at the start? Has assessment context been followed/applied in the HRA process?			
Dose/exposure—response relationship		Is the applicability of the selected dose/exposure - response relationship for the assessment thoroughly discussed?			
Exposure assessment (specific activities during which a specified number of individuals are exposed knowingly or unknowingly to the hazardous substances, location and characteristics of the location where the activities takes place, duration of activities, reason for activities, sources causing the presence of hazardous material in the environment where the activities take place...)	Exposure setting	Are the characteristics of the place of exposure clearly described?			
	Exposure sources	Are the major sources of hazardous material and/or activities causing the release(s) of hazardous material(s) into the environment identified?			
	Exposure duration	Is the duration and frequency of the exposure identified?			
	Exposed population	Is it clear who is really exposed (population/individuals, their number), and why are they exposed (e.g. their activities leading to exposure)?			
Magnitude of risk (is risk assessment explicitly determining the size of undesired effects)		Are the types of the expected adverse outcomes, their severity and the probability of their occurrence identified clearly?			
Uncertainty of HRA results		Are the major sources of uncertainty evaluated?			
Options for mitigating/avoiding exposure (specific options for mitigating exposure that are consistent and explicitly connected with the assessment context)		Are there any specific actions for avoiding or mitigating the exposure to the selected hazardous materials identified and/or proposed?			
Transparency and clarity of the assessment process		Is it transparent and clear how was the assessment performed and its conclusions obtained?			
answer with "Yes" if the publication demonstrates that the HRA element has been clearly shown ("No" if it does not)**<br>*mark with "X" only if it is clearly demonstrated that HBM is used in specific element (otherwise leave the column blank)**					

Appraisal comments

Publication title (citation)	Assessment context of HRA	Dose/exposure – response	Exposure setting	Exposure sources	Exposure duration	Exposed population	Magnitude of risk	Uncertainty of HRA results	Options for mitigating exposure	Transparency and clarity
Biomonitoring and health risks assessment of trace elements in various age- and gender-groups exposed to road dust in habitable urban-industrial areas of Hefei, China *(Ali et al., 2019)*	The study highlights what it set out in the title, namely the study of traces of elements in the nails and hair of those in a specific area of China. However, it did not carry out an in-depth analysis of the causes of the risks, focusing only on one element, which is the anthropogenic activity in the area and the consequent release of high amounts of elements harmful to human health. The other possible causes of the results collected (lifestyle of the people in the cohort, nutrition, occupation, possible long-term exposure, duration of exposure, among others) were only hinted at and not carefully analyzed. Biomonitoring through the analysis of biological material was carried out adequately. The use of nails to detect the traces of the elements is interesting, even if non-canonical.	Some factors were attributed to the high levels of these trace elements, but the issue of frequency to exposure was not addressed.	/	Only one of the possible causes from which all the elements described in the study come was extensively treated and analyzed (industrial activities). Many others were only hinted at but not addressed with the depth they deserve.	The issue of frequency of exposure is not addressed.	/	Adverse outcomes from exposure to a high amount of the treated elements are clarified and specified. The severity is also well-specified. The element that is not treated is the frequency of occurrence.	The major causes of uncertainty are only slightly determined, never going into specifics. For the analysis that the authors had set out to carry out, it would have been appropriate to spend more space on the treatment of the major causes of uncertainty in this type of study.	The authors suggest a campaign for the designation of new legislative measures. Surely, this would mitigate exposure to the treated elements, but there could be several other actions that could be taken to reduce the problem (awareness campaigns, re-qualification of the treated areas, etc.).	The presentation of the results and conclusions is clear, even if insufficient to be able to make a complete risk assessment in the treated population.

Publication title (citation)	Assessment context of HRA	Dose/exposure – response	Exposure setting	Exposure sources	Exposure duration	Exposed population	Magnitude of risk	Uncertainty of HRA results	Options for mitigating exposure	Transparency and clarity
Health risk assessment of trace metals through breast milk consumption in Saudi Arabia *(Al-Saleh, 2021)*	The study clearly followed its primary goal. It includes many different factors for the risk analysis of the elements analyzed, but lacks some important ones, as also reported in the dedicated section: for example, the dietary habits of mothers, the duration of lactation, and the average consumption of milk by infants per day and per week. In a study in which breast milk is analyzed, these are elements to be taken into consideration for a correct risk analysis. Other elements were excluded from the analysis, such as the uptake of substances from other sources and the cross-contamination of different substances.	An important element that is missing is the frequency of the babies' exposure.	The setting is only hinted at, but it is not specified.	The sources that can release the elements are well described, but they are insufficient for a complete risk analysis, as previously reported.	This is the study's biggest weakness, as previously reported.	/	All the elements described in the study are adequately treated with respect to the magnitude of the risk. As already mentioned, other elements had to be taken into consideration.	All weaknesses related to this study were reported by the authors in a specific section.	No solution to mitigate the problem has been addressed.	The presentation is clear and transparent. The conclusions regarding the results of the factors considered were treated adequately and clearly. However, the authors were well aware of the limitations of their study and the difficulty in finding an applicable solution to mitigate the problem.

Publication title (citation)	Assessment context of HRA	Dose/exposure – response	Exposure setting	Exposure sources	Exposure duration	Exposed population	Magnitude of risk	Uncertainty of HRA results	Options for mitigating exposure	Transparency and clarity
Exposure levels, determinants and risk assessment of organophosphate flame retardants and plasticizers in adolescents (14-15 years) from the Flemish Environment and Health Study *(Bastiaensen et al., 2021)*	The assessment clearly identifies that different flame retardants in human samples were analyzed and measured. However, I am not sure whether they applied the assessment context in the HRA process; yes for the first question, not sure for second question.	/	/	Temperature, season change, and use of household items were clearly explained and collected from each individual.	As they collected spot urine samples for analysis, they were unable to determine the duration of exposure.	It was not quite clear, as there can be different sources of exposure that were not taken into account in this study, such as dermal, inhalation, etc.	/	/	/	/

Publication title (citation)	Assessment context of HRA	Dose/exposure – response	Exposure setting	Exposure sources	Exposure duration	Exposed population	Magnitude of risk	Uncertainty of HRA results	Options for mitigating exposure	Transparency and clarity
Organophosphate pesticide exposure in children in Israel: Dietary associations and implications for risk assessment (Berman et al., 2020)	The risk assessment is only based on the comparison of HBM-derived exposure estimates with ADI.	/	A dietary interview was used for supporting information.	"It is possible that the higher concentrations of urinary DAP metabolites found in this population reflect relatively high consumption of fresh, regionally produced fruits and vegetables in the Israeli population, although we note there is a lack of published data in children" "In addition, it is possible that the higher concentrations of DM metabolites in this population reflect differences in agricultural use of OP pesticides."	/	"The sample of children in the RAV-MABAT survey was based on a random sample of children ages 2-11 years, from the population registry" and may not represent the entire population of children in Israel. "The sample of 100 children ages 4-11 years was designed to include children from different ethnic and geographic subgroups (girls/boys; urban/rural; Jewish/Arab) by defining specific quotas for different sectors and genders. We recruited children from the RAV-MABAT study to the current study until we filled these quotas, resulting in a total of 103 children that provided urine for the current study."	/	/	"These findings support regulatory action to reduce children's dietary exposure to OP pesticides, with regulatory options including phase out, additional restrictions on agricultural use of OP pesticides, and/or reduction of maximum permitted residue levels in produce frequently consumed by children."	/

Publication title (citation)	Assessment context of HRA	Dose/exposure – response	Exposure setting	Exposure sources	Exposure duration	Exposed population	Magnitude of risk	Uncertainty of HRA results	Options for mitigating exposure	Transparency and clarity
Exposure of Portuguese children to the novel non-phthalate plasticizer di-(iso-nonyl)-cyclohexane-1,2-dicarboxylate (DINCH) (Correia-Sá et al., 2017)	The study primarily assesses exposure to DINCH in a group of 112 children in Portugal. "For risk assessment we can either compare urinary metabolite levels or extrapolated daily intakes with health based guidance values."	/	/	/	/	/	/	/	"We can also advise exposure reduction measures if exposure trends indicate reaching critical levels, e.g., when exposure would approach TDI or RfD values, or if new toxicological findings lead to a reevaluation of health-based limit values."	/

Publication title (citation)	Assessment context of HRA	Dose/exposure – response	Exposure setting	Exposure sources	Exposure duration	Exposed population	Magnitude of risk	Uncertainty of HRA results	Options for mitigating exposure	Transparency and clarity
Exposure and risk assessment of Hg, Cd, As, Tl, Se, and Mo in women of reproductive age using urinary biomonitoring (Coscollà et al., 2021)	While risk assessment is one of the main three objectives, it is not clear why it is even performed–what concerns etc. is it addressing. The study is an HBM exposure assessment study that performs "risk assessment" only by calculation of HQ.	/	Only indirectly through questionnaire information.	Only indirectly through questionnaire information: "predictors of exposure."	/	"Mothers who had given birth at the University and Polytechnic Hospital La Fe in Valencia, Spain, between June and December 2015 were asked to participate in the study." The study limitations recognize that the selected study population cannot represent the entire population.	HQ is the selected measure of risk.	/	/	/

Publication title (citation)	Assessment context of HRA	Dose/exposure – response	Exposure setting	Exposure sources	Exposure duration	Exposed population	Magnitude of risk	Uncertainty of HRA results	Options for mitigating exposure	Transparency and clarity
Exposure and risk assessment of the Czech population to chlorinated pesticides and polychlorinated biphenyls using archived serum samples from the period 1970 to 1990 (Černá et al., 2016)	/	/	/	/	/	/	/	/	/	/

Publication title (citation)	Assessment context of HRA	Dose/exposure – response	Exposure setting	Exposure sources	Exposure duration	Exposed population	Magnitude of risk	Uncertainty of HRA results	Options for mitigating exposure	Transparency and clarity
Risk assessment of deoxynivalenol in a high-risk area of China by human biomonitoring using an improved high throughput UPLC-MS/MS method (Deng et al., 2018)	The only thing that was estimated in this paper is a probable daily intake using one equation.	/	/	/	/	It is clear who is exposed, but not why.	/	/	/	/

Publication title (citation)	Assessment context of HRA	Dose/exposure – response	Exposure setting	Exposure sources	Exposure duration	Exposed population	Magnitude of risk	Uncertainty of HRA results	Options for mitigating exposure	Transparency and clarity
Risk assessment of exposure to phthalates in breastfeeding women using human biomonitoring (Dualde et al., 2020)	/	/	It is not a controlled study, so the exposure setting can only be estimated using questionnaire data data derived from HBM, which did not provide this information.	Again, only estimated via questionnaire data.	Only the frequency was estimated in the questionnaires, but it is well-known that self-provided information on consumption frequencies comes with very high uncertainties; the data were derived from HBM, which did not provide this information.	/	/	The focus is on analytics.	Not the focus of the paper, but associated sources are named, so from those you could take this information. The data are derived from HBM, which did not provide this information.	/

Publication title (citation)	Assessment context of HRA	Dose/exposure – response	Exposure setting	Exposure sources	Exposure duration	Exposed population	Magnitude of risk	Uncertainty of HRA results	Options for mitigating exposure	Transparency and clarity
Evaluation of human biomonitoring data in a health risk based context: An updated analysis of population level data from the Canadian Health Measures Survey (Faure et al., 2020)	Not an actual HRA: "The study aims to provide an updated interpretation of population level CHMS biomonitoring data in a health risk based context using a set of biomonitoring screening values in order to identify chemicals for which current levels of exposure could be a concern." It continues the "risk screening" with HBM data and updated biomonitoring screening values.	Rsd only for cancer risk assessment (Cancer risk = ([biomarker] / BErsd) x 10−4), linear extrapolation assumed, no discussion about its applicability; biomarker values used are based on calculated P5, P25, P50, P75 and P95.	/	/	/	CHMS biomonitoring data are meant to be representative of the general population; why they are exposed is not identified.	Non-cancer effects only by comparisons with guidance values–HQ (BEs, RfD, TDI, etc.), while cancer risks are calculated with RSD (10-4 etc.) for different percentiles, e.g., "inorganic arsenic, cancer risk estimates were above the negligible risk range at all percentiles of the population assessed (ranging from 1.4×10−4 at P5, flagged for high variability to 1.4×10−3 at P95)."	They did not know how to interpret risks from some substances measured by HBM, or differences in BE confidence from one substance to another.	They focus on the identification of chemicals that require further evaluation.	Not actually HRA; however, the paper itself is clear.

Publication title (citation)	Assessment context of HRA	Dose/exposure – response	Exposure setting	Exposure sources	Exposure duration	Exposed population	Magnitude of risk	Uncertainty of HRA results	Options for mitigating exposure	Transparency and clarity
Biomonitoring of non-persistent pesticides in urine from lactating mothers: Exposure and risk assessment (Fernández et al., 2020)	The "aim of the present study was to assess the exposure to pesticides in urine from Spanish lactating mothers" and to "to perform a risk assessment for the most frequently detected pesticides"– the HRA process is degraded to a way of interpreting their results.	/	/	Only indirectly through predictors of exposure.	/	The representativeness of the study population is not discussed.	"Estimated daily intake (EDI), hazard quotient (HQ), and hazard index (HI) were obtained." "Regarding HQP95 values for OPs and deltamethrin (Table 5), all the obtained values were below one, and therefore, no risk existed for the Spanish mothers."	/	/	/

Publication title (citation)	Assessment context of HRA	Dose/exposure – response	Exposure setting	Exposure sources	Exposure duration	Exposed population	Magnitude of risk	Uncertainty of HRA results	Options for mitigating exposure	Transparency and clarity
Children's exposure to polycyclic aromatic hydrocarbons in the Valencian Region (Spain): Urinary levels, predictors of exposure and risk assessment (Fernández et al., 2021)	/	/	Map with the location of the schools, locations classified as urban or rural.	/	/	/	Hazard quotients and hazard indexes were estimated to assess the risk associated with PAH.	/	In the study, no hazardous exposure to PAH was observed, although concern was shown due to the high urine concentration of one of the metabolites compared to other countries; thus, further studies were suggested.	/

Publication title (citation)	Assessment context of HRA	Dose/exposure – response	Exposure setting	Exposure sources	Exposure duration	Exposed population	Magnitude of risk	Uncertainty of HRA results	Options for mitigating exposure	Transparency and clarity
Evaluation of exposure to phthalate esters and DINCH in urine and nails from a Norwegian study population (Giovanoulis et al., 2016)	/	/	It was not a controlled study, so the exposure setting could only be estimated using questionnaire data data derived from HBM, which did not provide this information.	Again, only estimated via questionnaire data.	Only the frequency was estimated in the questionnaires, but it is well known that self-provided information on consumption frequencies comes with very high uncertainties. The data were derived from HBM, which did not provide this information.	/	/	/	Associations with questionnaire data provide information on potential sources, so indirectly, this information is provided as data derived from HBM, which did not provide this information.	/

Publication title (citation)	Assessment context of HRA	Dose/exposure – response	Exposure setting	Exposure sources	Exposure duration	Exposed population	Magnitude of risk	Uncertainty of HRA results	Options for mitigating exposure	Transparency and clarity
Wastewater-based epidemiology for tracking human exposure to mycotoxins (Gracia-Lor et al., 2020)	Overall, the study mainly investigated the development of a new tool (WBE) to assist in future HRA for mycotoxins instead of actually completing an HRA themselves.	/	Only reference exposure via diet.	/	/	Wastewater analysis of mycotoxin metabolites for HBM.	/	/	/	/

Publication title (citation)	Assessment context of HRA	Dose/exposure – response	Exposure setting	Exposure sources	Exposure duration	Exposed population	Magnitude of risk	Uncertainty of HRA results	Options for mitigating exposure	Transparency and clarity
Biomonitoring of polychlorinated dibenzo-p-dioxins (PCDDs), polychlorinated dibenzofurans (PCDFs) and dioxin-like polychlorinated biphenyls (dl-PCBs) in human milk: Exposure and risk assessment for lactating mothers and breastfed children from Spain (Hernández et al., 2020)	It is primarily an exposure assessment study that compares the results with guidance values (TWI).	If TWI is regarded as a dose-response indicator.	/	/	/	The representativeness of the study population was not discussed.	/	Only of the study itself and not the HRA results.	/	/

Publication title (citation)	Assessment context of HRA	Dose/exposure – response	Exposure setting	Exposure sources	Exposure duration	Exposed population	Magnitude of risk	Uncertainty of HRA results	Options for mitigating exposure	Transparency and clarity
Predicted mercury soil concentrations from a kriging approach for improved human health risk assessment (Imo et al., 2018)	Using a kriging approach to improve the risk analysis for an element (Hg) in a specific restricted area (a region of Switzerland) could be an additional useful tool. A kriging-based prediction model could offer a very good visual of the population risk in a single area when integrated with specific parameters, such as geochemical data and population data, among others. This study analyzes a very large number of environmental samples and integrates them with other information from the population. Probably, for a complete model analysis, other factors should be added (e.g. cross-contamination, duration and frequency of exposure, among others). Furthermore, it would be congenial to include adverse effects and duration and frequency of exposure on the population in the model. It may be difficult to translate this model into a broader scenario where the variables to be taken into consideration are much greater. Either way, they hit the target they set out in the beginning.	The aim of the study was to verify whether it was useful to use information from a specific geostatistical analysis for an environmental health study. The dose/exposure-response relationship was barely or not taken into account, as were the duration and frequency of exposure.	The setting was well-described.	The possible main sources for Hg uptake were analyzed into three tables within the study. The authors included data from other works.	As mentioned before, duration and exposure to Hg were not considered.	The number of people in the cohort was well-described. The study was commissioned from a specific organization to assess potential health risks for resident populations.	A variety of information from the population was added to the model. A large number of different sources that could contribute to the risk of mercury uptake in the population were considered. The negative effects that the population can suffer and the severity and likelihood of them occurring were mentioned, but they must be primary factors to be taken into consideration.	The sources of uncertainty have been discussed previously and have not been taken into account in the proposed model.	No solution has been proposed to mitigate the problem of the risk of Hg exposure on the population. In any case, that was not the goal of the study.	This model tool certainly needs to be refined, but it is a great starting point and takes into account a wide range of variables for risk assessment in the population of a specific territory. As I said before, it may be difficult to translate this model into a broader scenario where the variables to be taken into consideration are much greater.

Publication title (citation)	Assessment context of HRA	Dose/exposure – response	Exposure setting	Exposure sources	Exposure duration	Exposed population	Magnitude of risk	Uncertainty of HRA results	Options for mitigating exposure	Transparency and clarity
Lead and mercury levels in repeatedly collected urine samples of young children: A longitudinal biomonitoring study (Kim et al., 2020)	This was an exposure assessment study (based on urine samples); the proportion of children at risk was evaluated by comparing values to German biomonitoring I and II and the reference value derived from the 95th percentile of representative samples. The "proportion of children at risk for Hg at each monitoring time point was evaluated based on the HBM I (7 μg/L) and II (25 μg/L), which were health-based guidance values derived on the basis of toxicological and epidemiological studies (Schulz et al., 2007) and the RV95 for German children (3-14 years) without dental amalgam fillings (0.4 μg/L) (Apel et al., 2017). However, the proportion of children at risk for Pb based on HBM values was not evaluated, because the corresponding HBM I and II were not available, while the proportion of children at risk for Pb was evaluated based on the RV95 for Canadian children (3-5 years) (1.7 μg/L) (Saravanabhavan et al., 2017)."	/	/	"It was not possible to find the environmental causes of the Pb and Hg exposures in children of our study due to the limited information for exposure sources."	/	The "target population of the present study was general Korean children aged 0-27 month(s) who were born healthy." Representativeness was not known.	Only estimated proportion of children at risk (levels of Hg/Pb over RV95).	Within the study scope.	/	/

Publication title (citation)	Assessment context of HRA	Dose/exposure – response	Exposure setting	Exposure sources	Exposure duration	Exposed population	Magnitude of risk	Uncertainty of HRA results	Options for mitigating exposure	Transparency and clarity
Exposure to the plasticizer di(2-ethylhexyl) terephthalate (DEHTP) in Portuguese children - Urinary metabolite levels and estimated daily intakes (Lessmann et al., 2017)	/	/	/	/	The duration and frequency are not explicitly identified, but since the exposure route is oral, it can be considered that the study investigates accumulated exposure based on the health habits of the participants.	/	/	The authors mentioned some of the uncertainty sources without much elaboration.	/	/

Publication title (citation)	Assessment context of HRA	Dose/exposure – response	Exposure setting	Exposure sources	Exposure duration	Exposed population	Magnitude of risk	Uncertainty of HRA results	Options for mitigating exposure	Transparency and clarity
Exposure and health risk assessment of secondary contaminants closely related to brominated flame retardants (BFRs): Polybrominated dibenzo-p-dioxins and dibenzofurans (PBDD/Fs) in human milk in Shanghai (Y. Lin et al., 2021)	Attempt at exposure assessment through HBM measurements (risk assessed only later– comparison with TDI).	Only indirectly through a comparison of EDI with TDI.	/	/	/	/	/	/	None apart from more research (including environmental measurements to determine the presence of PBDEs and non-PBDE BFRs in environments).	/

Publication title (citation)	Assessment context of HRA	Dose/exposure – response	Exposure setting	Exposure sources	Exposure duration	Exposed population	Magnitude of risk	Uncertainty of HRA results	Options for mitigating exposure	Transparency and clarity
Integration of biomonitoring data and reverse dosimetry modeling to assess population risks of arsenic-induced chronic kidney disease and urinary cancer (Y.-J. Lin et al., 2020)	/	/	/	/	/	/	/	/	/	/

Publication title (citation)	Assessment context of HRA	Dose/exposure – response	Exposure setting	Exposure sources	Exposure duration	Exposed population	Magnitude of risk	Uncertainty of HRA results	Options for mitigating exposure	Transparency and clarity
Exposure assessment of Portuguese population to multiple mycotoxins: The human biomonitoring approach (Martins et al., 2019)	This was an exposure assessment study, which includes PDI and HQ calculation (referred to as risk characterization) through reverse dosimetry.	/	/	Assumption that contaminated food is the main source,	Urine samples connected with fast excretion rates.	It targets the Portuguese population, but it is not clear if 94 participants are representative.	/	Missing data, knowledge (mycotoxin metabolism); data derived from animal studies.	More studies are needed.	However, it is an exposure assessment more than an actual HRA.

Publication title (citation)	Assessment context of HRA	Dose/exposure – response	Exposure setting	Exposure sources	Exposure duration	Exposed population	Magnitude of risk	Uncertainty of HRA results	Options for mitigating exposure	Transparency and clarity
Glyphosate in Portuguese adults– A pilot study (Nova et al., 2020)	An "exploratory glyphosate exposure assessment was conducted among Portuguese adults."	Exposure compared to EFSA's ADI, AOEL.	/	"Environmental exposure (such as food or water intake) and occupational settings seem to be the main routes for glyphosate contamination."	Two sampling: "chronic exposure is a reality."	"Self-selected participants."	"Systematically available internal doses values were below EFSA's risk assessment values (ADI or AOEL), and as such, the concentration values measured in this study are not per se a human health problem."	Within the study scope.	/	A proper HRA was not performed.

Publication title (citation)	Assessment context of HRA	Dose/exposure – response	Exposure setting	Exposure sources	Exposure duration	Exposed population	Magnitude of risk	Uncertainty of HRA results	Options for mitigating exposure	Transparency and clarity
Exposure of nursing mothers to polycyclic aromatic hydrocarbons: Levels of un-metabolized and metabolized compounds in breast milk, major sources of exposure and infants' health risks (Oliveira et al., 2020)	/	/	/	/	/	They specify that lactating/pregnant females are getting exposed and the concentration inside females is variable due to metabolic activity. They suggest that this exposure may be due to eating habits and environmental/residential exposure during breastfeeding.	/	Uncertainty related to exposure was mentioned but not evaluated clearly.	/	/

Publication title (citation)	Assessment context of HRA	Dose/exposure – response	Exposure setting	Exposure sources	Exposure duration	Exposed population	Magnitude of risk	Uncertainty of HRA results	Options for mitigating exposure	Transparency and clarity
Biomonitoring of mercury in hair of children living in the Valencian Region (Spain). Exposure and risk assessment (Pérez et al., 2019)	This "work reports the results of total mercury (THg) in hair samples of a representative population of children (6-11 years of age) from the Valencian Region (Spain) participating in the BIOVAL program. We also compare the results with similar national and international studies, and perform an analysis of the main predictors of exposure. The risk was characterized by comparing the levels of Hg in hair using international health-based guidance values."	/	/	Predictors: "Multiple regression analysis revealed that fish consumption, mother's country of birth (Spain or abroad) and the employment situation of parents were the main predictors of mercury in hair."	/	Study population: a representative population of children (6-11 years of age) from the Valencian Region (Spain) participating in the BIOVAL program.	"About 13% of children had hair mercury levels above the FAO/WHO JECFA guideline of 2.3 mg g 1 and 18% of children had levels above the EFSA health-based guidance value of 1.9 mg."	/	"This means that the measures to reduce Hg exposure, that predators, such as sword fish and tuna fish, have to be promoted with more effectiveness." "In this sense the Regional authorities of the Valencian Region recommend avoiding these fish species in school menus and promoting the consumption of fish with lower levels of Hg." They mainly focus on recommending a reduced consumption of big fish.	/

Publication title (citation)	Assessment context of HRA	Dose/exposure – response	Exposure setting	Exposure sources	Exposure duration	Exposed population	Magnitude of risk	Uncertainty of HRA results	Options for mitigating exposure	Transparency and clarity
Estimating human exposure to pyrethroids' mixtures from biomonitoring data using physiologically based pharmacokinetic modeling (Quindroit et al., 2021)	/	/	/	/	They considered continuous exposure, but it is not very clear.	/	/	/	/	/

Publication title (citation)	Assessment context of HRA	Dose/exposure – response	Exposure setting	Exposure sources	Exposure duration	Exposed population	Magnitude of risk	Uncertainty of HRA results	Options for mitigating exposure	Transparency and clarity
Cadmium exposure in First Nations communities of the Northwest Territories, Canada: smoking is a greater contributor than consumption of cadmium-accumulating organ meats (Ratelle, Li, et al., 2018)	/	Questionnaire about organ meat consumption, measurement of organ meats, and relation to blood and urine concentrations (Cd) of participants.	/	/	Reported on (yes/no) consumption in the past year. Perhaps not defined well enough?	/	Specific probabilities not given.	/	N/A = risk was deemed to be low/below critical thresholds for the action investigated, so it wasn't necessary to provide mitigating recommendations.	/

Publication title (citation)	Assessment context of HRA	Dose/exposure – response	Exposure setting	Exposure sources	Exposure duration	Exposed population	Magnitude of risk	Uncertainty of HRA results	Options for mitigating exposure	Transparency and clarity
Implementation of human biomonitoring in the Dehcho region of the Northwest Territories, Canada (2016-2017) (Ratelle, Skinner, et al., 2018)	/	/	/	/	/	/	/	/	/	/

Publication title (citation)	Assessment context of HRA	Dose/exposure – response	Exposure setting	Exposure sources	Exposure duration	Exposed population	Magnitude of risk	Uncertainty of HRA results	Options for mitigating exposure	Transparency and clarity
Assessment of human exposure to selected pesticides in Norway by wastewater analysis (Rousis et al., 2020)	Similarly to Giovanoulis et al. 2016, this study was not really conducting an HRA itself (more of an exposure assessment) but was investigating new techniques to use in future risk assessments. There was no relevant assessment endpoint.	The study assessed a panel of chemicals.	/	/	/	HBM using wastewater for metabolites.	/	/	/	/

Publication title (citation)	Assessment context of HRA	Dose/exposure – response	Exposure setting	Exposure sources	Exposure duration	Exposed population	Magnitude of risk	Uncertainty of HRA results	Options for mitigating exposure	Transparency and clarity
Biomonitoring of bisphenols A, F, S and parabens in urine of breastfeeding mothers: Exposure and risk assessment (Sanchis et al., 2020)	/	/	/	/	/	/	/	/	/	/

Publication title (citation)	Assessment context of HRA	Dose/exposure – response	Exposure setting	Exposure sources	Exposure duration	Exposed population	Magnitude of risk	Uncertainty of HRA results	Options for mitigating exposure	Transparency and clarity
Integrated exposure and risk characterization of bisphenol-A in Europe (Sarigiannis et al., 2016)	No clear assessment context was identified. The study was about an approach that estimates exposure based on multiple data available (HBM, food concentrations, etc.) and proposed hypothetical exposure scenarios: the "study aims to quantify external and internal exposure to BPA and to assimilate biomonitoring data in Europe using the integrated modelling framework of the INTEGRA computational platform."	Dose-responses only considered in the development of some dose estimates (as parameters of the PBTK model).	There was no real identification of the exposure setting, only indirect/hypothetical proposal of exposure scenarios; HBM data were used for improving external exposure estimates.	There was no real identification of sources that would be assessment-specific (a specific real population actually exposed to something), and the study was only based on indirect/hypothetical proposal of exposure scenarios (e.g., using data from different studies, finding BPA in canned food, formula, beverages...).	There was no real identification of exposure duration, only indirect/hypothetical proposal of exposure scenarios; HBM data were used for improving external exposure estimates.	The population is only hypothetical (an actual, real population is not determined; the applicability of the study for a specific population not discussed); exposure scenarios are proposed but not verified. Premature neonates in intensive care units are identified as the most vulnerable BPA population group.	Risk characterization was carried out by comparing exposure information (modelled) with TDI, BE, RfD, MRLs; exposure scenarios that might pose a public health risk were proposed: "we concluded that exposure to BPA does not pose any significant threat according to most realistic exposure scenarios."	Inherent uncertainties of parameters of the BPA toxicokinetics.	/	Multiple data sources were used for the modelling, but their applicability/reliability is not individually evaluated or discussed. The study was considered an "initial point for a more comprehensive interaction among investigators of different disciplines, bridging exposure scenarios, biomonitoring, toxicological and epidemiological data iteratively in order to identify and fill the current knowledge gaps."

Publication title (citation)	Assessment context of HRA	Dose/exposure – response	Exposure setting	Exposure sources	Exposure duration	Exposed population	Magnitude of risk	Uncertainty of HRA results	Options for mitigating exposure	Transparency and clarity
Risk characterization of bisphenol-A in the Slovenian population starting from human biomonitoring data (Sarigiannis et al., 2019)	It was clearly mentioned in the abstract that RCR values with respect to TDI set by EFSA were found to be less, which indicates that there is minimum risk.	/	/	/	/	/	/	/	/	/

Publication title (citation)	Assessment context of HRA	Dose/exposure – response	Exposure setting	Exposure sources	Exposure duration	Exposed population	Magnitude of risk	Uncertainty of HRA results	Options for mitigating exposure	Transparency and clarity
Human biomonitoring in urine samples from the Environmental Specimen Bank reveals a decreasing trend over time in the exposure to the fragrance chemical lysmeral from 2000 to 2018 (Scherer et al., 2021)	/	/	The substance is present in daily care products; therefore, exposure is not associated with a particular place or activity.	/	The substance is present in cosmetics, detergents, and other products with fragrance. Therefore, exposure to the compound is expected to be frequent when encountered. Time points of the collection of samples were specified (years), since the main aim of the study is to determine the temporal trend of exposure	/	There is not enough available data on the effects of the compound (lysmeral) to describe the severity and probability of adverse outcomes.	24h urine volume was preferentially used for normalization since creatinine can add variability due to confounding factors such as body weight. There was an identification of the best metabolite as biomarker (TCBB and lysmerol) for lysmeral exposure through correlation. The study population is not representative of the general population (demographics).	Further studies suggested with a broader population and aggregate exposure of synthetic chemicals. No specific actions for mitigating exposure suggested. The levels found in urine were much lower than those in DNELs.	/

Publication title (citation)	Assessment context of HRA	Dose/exposure – response	Exposure setting	Exposure sources	Exposure duration	Exposed population	Magnitude of risk	Uncertainty of HRA results	Options for mitigating exposure	Transparency and clarity
Bisphenol A and six other environmental phenols in urine of children and adolescents in Germany - human biomonitoring results of the German Environmental Survey 2014-2017 (GerES V) (Tschersich et al., 2021)	/	/	/	/	/	/	/	/	/	/

Publication title (citation)	Assessment context of HRA	Dose/exposure – response	Exposure setting	Exposure sources	Exposure duration	Exposed population	Magnitude of risk	Uncertainty of HRA results	Options for mitigating exposure	Transparency and clarity
Multicenter biomonitoring of polybrominated diphenyl ethers (PBDEs) in colostrum from China: Body burden profile and risk assessment (Yin et al., 2019)	A multicenter study was conducted in three different Chinese cities: Mianyang, Wuhan and Hangzhou, representing western, central and eastern China, respectively. Colostrum samples were collected from local residents. This study was intended to: 1) determine and compare the concentration levels and patterns of tri-to hexa-BDEs in colostrum samples from different cities, 2) investigate the influencing factors on the PBDE concentrations, 3) evaluate the association between the maternal exposure of PBDEs and birth outcomes, and 4) provide information on the health risks associated with breastfeeding in the first week of infant's life. (HQ; comparisons with EDI).	HQ calculation.	/	Indirectly through questionnaire responses and assumptions.	/	Study population: "Eligibility criteria included residing in the sampling cities for at least five years, no clinical or historical maternal illnesses, and a singleton birth baby. A total of 60 mothers voluntarily participated, and from each city, 20 participants were recruited." Not representative.	"In this study, the potential health risk of PBDEs for infants via breastfeeding was assessed using hazard quotients (HQs) on the basis of the congener specific reference doses (RfD) of BDE-47, BDE-99, and BDE-153. The HQs of BDE-153 in 24 colostrum samples and BDE-47 in 2 samples were higher than 1 (Fig. 2). The results showed potential chronic hazard for future exposure to PBDE congeners. Neonates from Mianyang are highly exposed to BDE-153 via breastfeeding. The HQ of BDE-153 for 80% (16 of 20) of the babies from Mianyang was higher than 1. For the THQ, 60% of infants have an estimated value higher than 1, suggesting that ingestion of PBDEs caused potential risk for breastfed infants in this study." "Among all samples, 60% of infants have an estimated total hazard quotient>1, indicating a potential health risk from PBDE exposure through breastfeeding for the infants."	/	/	Sample conclusion: "Infant formulas seemed to have a lower risk for neonates than the colostrum, but the advantages in breastfeeding will outweigh the risks and potential adverse health effects caused by environmental PBDEs and other xenobiotic chemical exposure." This was not supported by observations or references.

Publication title (citation)	Assessment context of HRA	Dose/exposure – response	Exposure setting	Exposure sources	Exposure duration	Exposed population	Magnitude of risk	Uncertainty of HRA results	Options for mitigating exposure	Transparency and clarity
Biomonitoring and subsequent risk assessment of combined exposure to phthalates in Iranian children and adolescents (Jeddi et al., 2018)	HQ-based assessment of "risk." "The aim of the present study was to determine the extent of exposure to the phthalates BBP, DBP, DEHP, DEP and DMP for the first time among children and adolescents in Iran, and to estimate for this population the risk of exposure to the individual phthalates as well as to the combined exposure to the anti-androgenic phthalates BBP, DBP, DEHP." "Hazard quotients (HQs) were calculated for quantifying potential risks for children and adolescents from exposure to single phthalates, which are defined as the ratio between the EDI and their health-based guidance values and/or acceptable level of exposure listed in Table 2 and comprising the Tolerable Daily Intake (TDI), the Reference Dose (RfD) and the Reference Dose for Anti-Androgenicity (RfD-AA) established by the EFSA, U.S. EPA and Kortenkamp and Faust (2010) [62]," "To assess combined exposures to multiple phthalates, the Hazard Index (HI) was used, which is based on the dose addition concept."	There was some discussion of the applicability of the EFSA's TDI, the EPA's RfD included, but not in detail.	/	/	/	Study population: 56 healthy children and adolescents ranging from 6 to 18 years of age in Tehran, Iran (between September and November 2015). "Random house addresses for recruiting participants and collecting urinary samples were selected by a weighted approach based on Tehran's population density using ArcGIS software." Representativeness was not discussed.	"The data indicated that the Iranian children and adolescents were exposed to a mixture of phthalates, and a subsequent risk assessment revealed that none of the surveyed participants had HQ and HI values that raised a concern."	/	/	HBM-based exposure assessment calculating HI, HQ.

Publication title (citation)	Assessment context of HRA	Dose/exposure – response	Exposure setting	Exposure sources	Exposure duration	Exposed population	Magnitude of risk	Uncertainty of HRA results	Options for mitigating exposure	Transparency and clarity
Antibiotic body burden of elderly Chinese population and health risk assessment: A human biomonitoring-based study (Zhu et al., 2020)	"This is the first study to examine the exposure of an elderly cohort to antibiotics using bio-monitoring. Here, we monitored 45 antibiotics and two antibiotic metabolites in urine samples of approximately 1,000 elderly individuals from Lu'an city of West Anhui province, China and assessed health risks due to antibiotics among them based on microbiological effects." "On the basis of EDI estimations, we further calculated hazard quotient (HQ) and hazard index (HI) to assess health risks from a single antibiotic exposure and combined exposure, respectively."	/	/	Indirectly.	/	"However, the large sample size used in this study may reflect the average exposure of China's elderly population to antibiotics."	Partially: "Of the detected antibiotics, three tetracyclines, six fluoroquinolones, and three other antibiotics (trimethoprim, thiamphenicol, and lincomycin) were found to pose a microbiological effect-related health risk in 6.7% of the elderly individuals, which was higher than 4.3% in pregnant women (Wang et al., 2017b) and 6.0% in children (Wang et al., 2018a), but lower than 7.2% in general adults."	/	"Therefore, the government should formulate ciprofloxacin usage guidelines for humans and animals."	/
